# Probiotics: functional food ingredients with the potential to reduce hypertension

**DOI:** 10.3389/fcimb.2023.1220877

**Published:** 2023-07-03

**Authors:** Zouquan Chen, Wanjie Liang, Jie Liang, Jiaxin Dou, Fangyu Guo, Daolei Zhang, Zhenshang Xu, Ting Wang

**Affiliations:** ^1^ State Key Laboratory of Biobased Material and Green Papermaking, Qilu University of Technology, Shandong Academy of Science, Jinan, China; ^2^ School of Bioengineering, Qilu University of Technology, Shandong Academy of Science, Jinan, China; ^3^ Research and Development Department(R&D), Shandong Ande Healthcare Apparatus Co., Ltd., Zibo, China; ^4^ School of Bioengineering, Shandong Polytechnic, Jinan, China; ^5^ Henan Province Key Laboratory of Water Pollution Control and Rehabilitation Technology, Henan University of Urban Construction, Pingdingshan, China

**Keywords:** probiotics, functional foods, hypertension, mechanisms, intestinal flora

## Abstract

Hypertension is an increasingly pressing public health concern across the globe. It can be triggered by a variety of factors such as age and diet, as well as the stress of modern life. The traditional treatment of hypertension includes calcium ion blockers, angiotensin II receptor inhibitors and β-receptor blockers, but these drugs have at least some side effects. Recent studies have revealed that intestinal flora plays a vital role in maintaining and promoting human health. This is due to the type and amount of probiotics present in the flora. Probiotics can reduce hypertension symptoms through four mechanisms: regulating vascular oxidative stress, producing short-chain fatty acids, restoring endothelial cell function, and reducing inflammation. It has been reported that certain functional foods, using probiotics as their raw material, can modify the composition of intestinal flora, thus regulating hypertension symptoms. Consequently, utilizing the probiotic function of probiotics in conjunction with the properties of functional foods to treat hypertension is a novel, side-effect-free treatment method. This study seeks to summarize the various factors that contribute to hypertension, the mechanism of probiotics in mitigating hypertension, and the fermented functional foods with probiotic strains, in order to provide a basis for the development of functional foods which utilize probiotics as their raw material and may have the potential to reduce hypertension.

## 1 Introduction

With the rapid development of social economy, the daily dietary structure has changed significantly, leading to an increasing prevalence of excess food nutrition. That, as well as the accelerated pace of life and work, has caused the age composition of hypertension patients to tend towards younger (Rison et al., 2022). Poor eating habits, fat accumulation, irregular work and rest, environmental stimulation (Park et al., 2020), and family genetics can all contribute to hypertension (Arnett and Claas, 2018). Hypertension management usually requires long-term treatment with drug combinations to control the disease. However, the long-term use of anti-hypertensive drugs may have a significant side effect on the human body, such as depression caused by the long-term use of β-receptor blockers (Zhang et al., 2022). Furthermore, there are various restrictions on the use of blood pressure medications in patients with diabetes, chronic kidney disease, and pregnancy. For example, angiotensin-converting enzyme inhibitors (ACEs) and angiotensin-receptor inhibitors (ARBs) should be discontinued immediately in pregnant women with hypertension (Wei et al., 2021).

In recent years, the rapid development of sequencing technology has enabled us to further explore and study the role of human microbial communities in our physiological health (Gebrayel et al., 2022). A significant association has been established between the composition of the gut microbiome and cardiovascular diseases, such as hypertension (Lakshmanan et al., 2021). Over the past decade, a significant body of evidence has emerged to support the notion that the gut microbiome can regulate blood pressure. In the past five years, the field has progressed from association to causation, as research has revealed various mechanisms by which the gut microbiome can regulate blood pressure (O’Donnell et al., 2023). These studies have uncovered a variety of mechanisms by which gut microbes regulate hypertension, ranging from “why gut microbes can affect hypertension” to “how gut microbes affect hypertension”. This has further opened up possibilities for new treatments which could potentially prevent hypertension by regulating the gut microbiome.

The Food and Agriculture Organization of the United Nations (FAO) and the World Health Organization (WHO) define probiotics as “live microorganisms that, when consumed in sufficient quantities, have a beneficial effect on the host” (Jäger et al., 2019). In recent years, probiotics have gained public attention for their potential to promote human health. This has led to a rapid expansion of the global probiotics market, resulting in the application of probiotics being utilized not just in supplements, but also as raw materials for functional foods. Probiotics are able to affect the body in multiple ways, such as through the secretion of metabolites which can directly impact other microorganisms and regulate the host defense system (De Vos et al., 2022). Furthermore, they can help to prevent invading pathogens, as well as target digestive functions and chronic inflammation (Cristofori et al., 2021). Additionally, probiotics have been studied for their potential anti-cancer properties (Eslami et al., 2019). More recently, research has focused on the potential of probiotics as functional raw materials for hypertension prevention.

Granato et al. (2020) defines functional foods as industrially processed or natural foods that, when consumed regularly in effective levels as part of a varied diet, may have a positive impact on health in addition to essential nutrients. However, in order for any functional food to be widely used, a large number of randomized, double-blind, controlled clinical trials must first be conducted to prove its safety and functionality. Therefore, no fresh, unprocessed or processed food can be considered a functional food without the proper evidence. Generally, functional foods can be integrated into a daily diet and contain bioactive ingredients which can reduce the risk of multiple diseases. For those with hypertension, consuming functional foods based on probiotics – which have not been found to have any side effects – can help to maintain a normal blood pressure range (Granato et al., 2020). In this study, we provided a theoretical basis for the daily treatment and prevention of hypertension by introducing the functional foods containing probiotics.

## 2 Hypertension and gut microbes

### 2.1 Hypertension

Hypertension, characterized by elevated arterial blood pressure and persistent high blood pressure, can lead to stroke, heart disease, kidney disease, and other derived diseases. It can be divided into primary and secondary hypertension (Rivas et al., 2021). Essential hypertension is an elevation in blood pressure that is not caused by any other disease, whereas secondary hypertension is an increase in blood pressure caused by other diseases.

**Figure 1:**
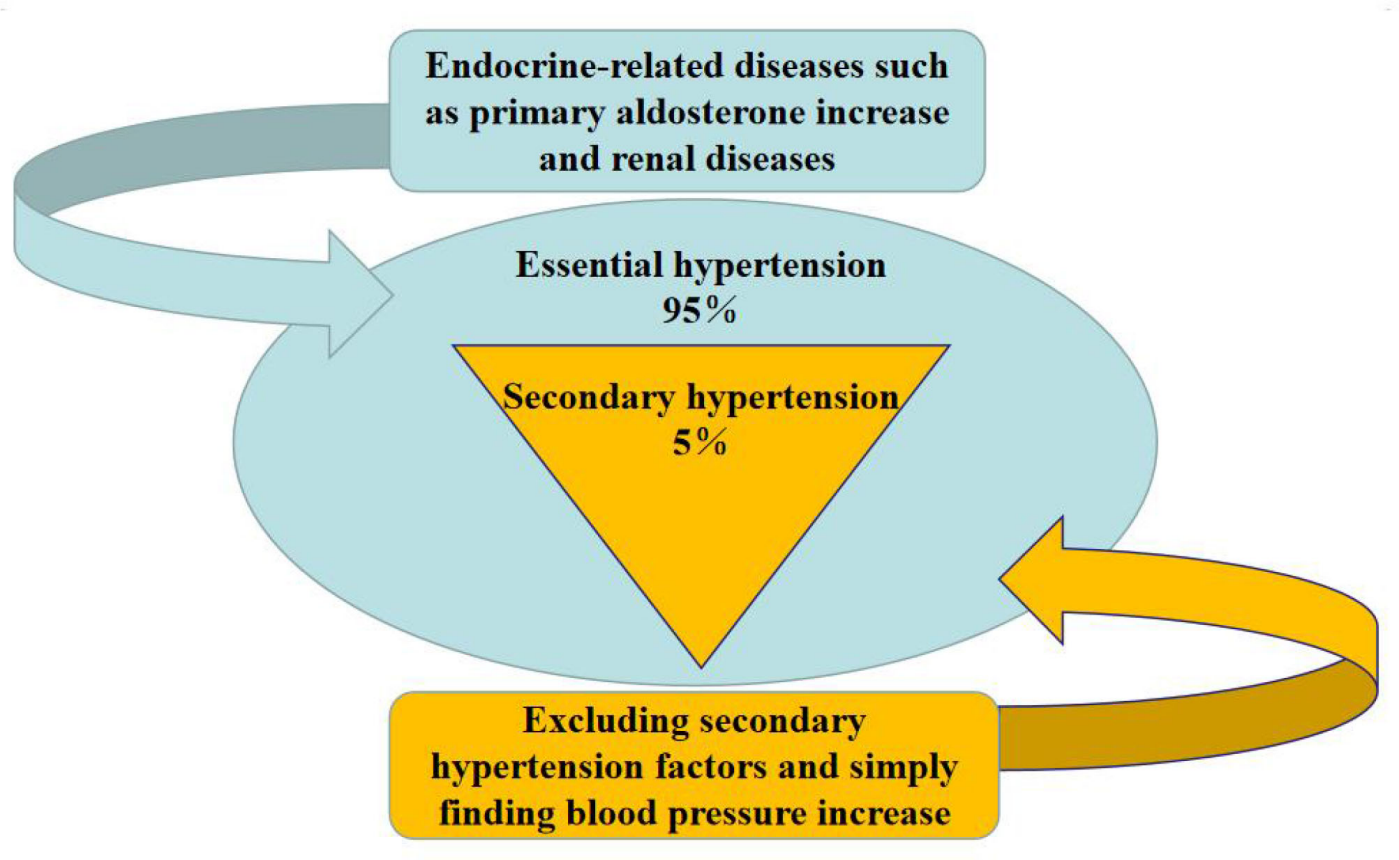
The types and causes of hypertension in patients.

As shown in **Figure 1**, the coverage of hypertension is extensive, as different groups present with differing symptoms and types. Approximately 20-30% of childhood hypertension is primary, while 65-80% is secondary. Hypertensive syndrome of pregnancy, also known as pregnancy poisoning or pre-eclampsia, is unique to pregnant women and typically appears around 20 weeks gestation or within two weeks postpartum (Ives et al., 2020). Senile systolic hypertension, which refers to systolic blood pressure higher than normal levels and normal diastolic blood pressure in elderly individuals over 60 years old, is an independent condition and a risk factor for senile vascular diseases and stroke (Hu et al., 2013).

### 2.2 Intestinal microorganisms

**Figure 2:**
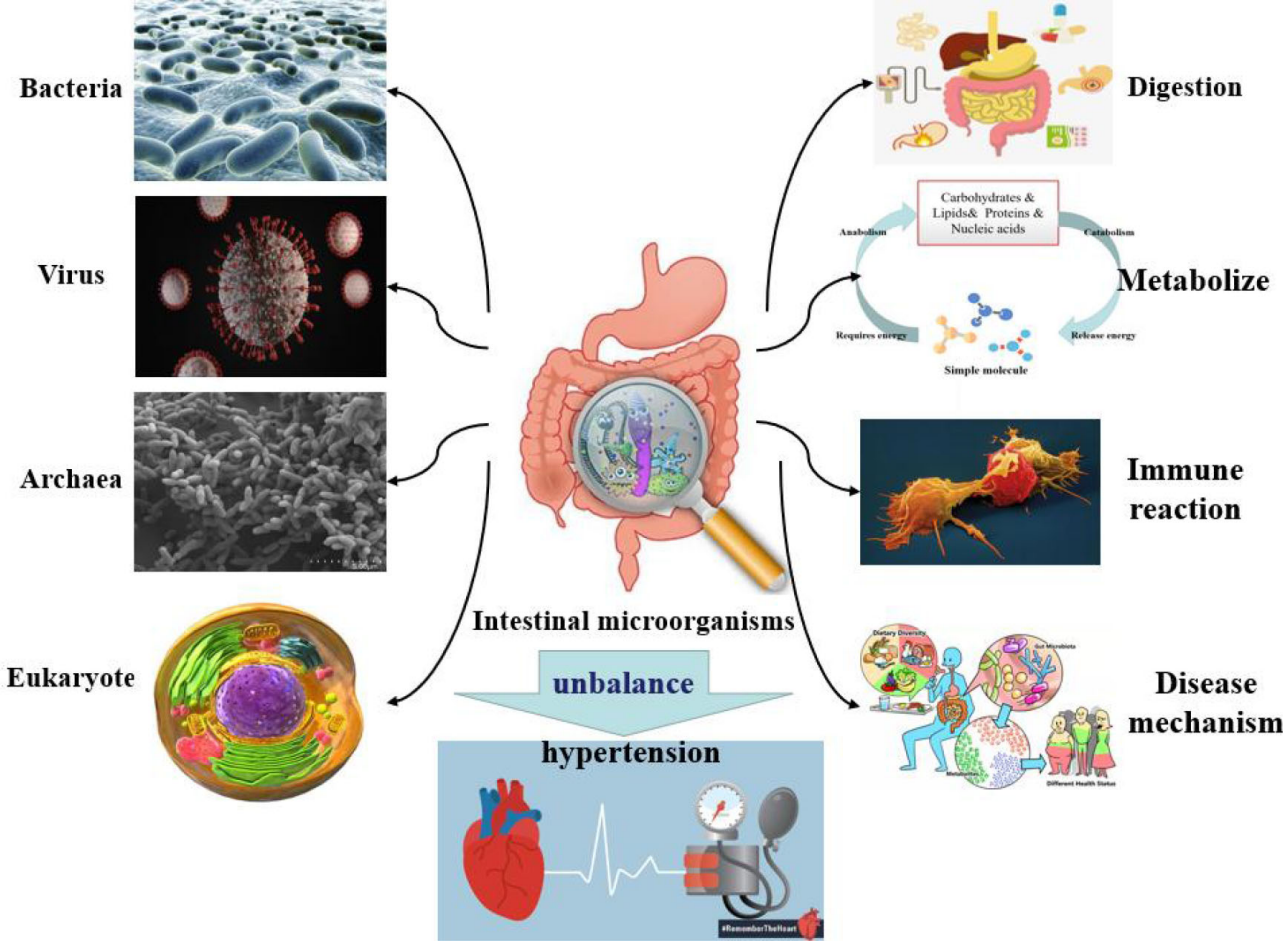
The composition of the microbiota and their physiological roles in the host.

The host microbiome is a complex ecosystem consisting of bacteria, eukaryotes, viruses, and archaea which coexist on tissue surfaces and within the body (**Figure 2**). These microbial communities play important roles in physiological activities such as digestion, metabolism, immune response, compound synthesis, toxin elimination, and even the pathogenesis of certain diseases (Gasaly et al., 2021). Intestinal microorganisms have been found to have a role in the occurrence and maintenance of hypertension. When researchers studied a model of spontaneous hypertension, they noted a series of changes in intestinal physiology, such as changes in intestinal microbes and the amount of tight junction proteins in the intestinal wall (Kim et al., 2018). The imbalance of intestinal microorganisms, combined with the increased inflammation, leads to abnormal sympathetic nerve and intestinal function, which can ultimately lead to the development of hypertension (Guyenet et al., 2020). Li et al. (2021) conducted bacterial genome analysis and fecal microbiome transplantation to find that an abnormal gut microbiota richness was significantly associated with hypertension. The research results of Liu et al. (2018) indicated that the number of *Bifidobacterium* and *Bacteroides* decreased, while the number of *Eubacillus rectalis* increased in the intestinal tract of patients with hypertension, and that the content of intestinal flora was correlated with blood pressure.

Hypertensive individuals and animals have been observed to have reduced bacterial diversity in their gut microbiome, disordered microbial structure and function, and the fermentation end products often altered (Li et al., 2021). This imbalance of intestinal flora and the change of metabolites are key factors in controlling blood pressure. With the development and widespread use of metagenomic sequencing and metabolomics, the relationship between metabolites of intestinal flora and human cardiovascular diseases has been brought to the attention of public. In particular, recent studies on metabolite have found that trimethylamino-N-oxide (TMAO) contributes to the promotion of atherosclerosis through the production of pro-inflammatory cytokines (TNF-α and IL-1B), the reduction of anti-inflammatory cytokines (IL-10), especially the excessive reaction of platelets led to the formation of thrombus (Livshits and Kalinkovich, 2019). Calderón-Pérez et al. (2020) studied gut microbes by targeting microbial metabolites short chain fatty acids (SCFA) and TMAO in plasma and stool. The results of their study showed that there were significant differences in the types and richness of intestinal flora between normal people and hypertensive patients.

Based on the microbiome and microbial metabolites, it is possible to distinguish individuals with hypertension from those without before treatment with drugs. Furthermore, this may be used to initially identify individuals with hypertension symptoms.

### 2.3 Probiotics and functional foods

Probiotics are live, non-pathogenic microorganisms that, when present in sufficient amounts, may confer health benefits on the host (Salminen et al., 2021). The application of probiotics is commonly achieved in the form of raw materials for functional foods (Rashidinejad et al., 2022). Functional foods are defined as food with specific nutritional and health-promoting functions, meant for certain people with the purpose of regulating body functions (Gul et al., 2016). These include foods for improving body constitution, preventing certain diseases (such as hypertension, diabetes, and coronary heart disease), and restoring health. However, functional foods differ from drugs in that they are not used for treatment, and cannot replace medication for patients. In addition, functional foods must be non-toxic or, at least, exhibit toxicity levels below the threshold set by modern toxicology, meaning that they pose no significant risk of toxic side effects with regular consumption. When compared to current antihypertensive drugs, functional foods can be used to relieve mild symptoms and prevent elevated blood pressure, thus alleviating or counteracting the disease without the risk of developing depression associated with β-receptor blockers (Yeo et al., 2009).

In recent years, probiotics have been proposed as an alternative to antimicrobials and as an adjunct therapy for various chronic diseases (Tsai et al., 2019). Microorganisms directly participate in the metabolic mechanisms of certain diseases, or produce metabolites that affect certain diseases (Hou et al., 2022), such as histone deacetylase inhibitor metabolites that modify cancer susceptibility and lesions. The metabolites produced by probiotics upon entering the body, commonly known as prebiotics, can have direct effects on atherosclerosis (Vourakis et al., 2021), hypertension (Muralitharan et al., 2020), heart failure (Kaye et al., 2020), Type-2 diabetes (Iatcu et al., 2021), and other diseases. Probiotics can directly or indirectly affect host physiology through the production of prebiotics, such as TMAO, SCFA, secondary bile acids and indoxyl sulfate. These metabolites can stimulate a variety of signaling pathways that control physiological processes.

Probiotics have become increasingly popular due to their beneficial properties as functional foods and are now frequently used in everyday life. Besides traditional frozen dairy products, bread and snack products provide new opportunities for the development of probiotics, such as probiotic nuts, probiotic wafer cookies launched in the Chinese market, and energy bars with probiotics added in the Australian market. In the past, probiotics were often fragile and had to be refrigerated, making them difficult to be incorporated into products other than dairy products. However, with the increasing demand, more and more tolerant probiotics have been screened out, such as adapting to extreme pH, high temperature, low temperature, or high pressure. This expands the possibilities for probiotics to be used in frozen foods, hot drinks, and snacks. The growing market of probiotic consumption further indicates people’s newfound understanding of probiotics. Japan, which was the first country to commercially utilize probiotics, has put a lot of emphasis on the probiotic market. Lactic acid drinks containing probiotics have been used in Japan since the 1970s, making it one of the largest consumers of probiotic products in the world. Meanwhile, some enterprises have also emerged in the probiotic market in other countries as listed in **Table 1**. These enterprises have a long history and rich experience in the production and processing of probiotics.

**Table 1:** The origin and establishment time of the representative probiotic product manufacturers, and their well-known products and the types of probiotics.

Brand	Origin	Establishment time	Well-known products	Related probiotics
Osteoform	USA	1995	HIGHLY ACTIVE PROBIOTICS	*Lactobacillus reuteri*
i-Health	USA	1995	Culturelle	*lacticaseibacillus rhamnosus*
Hanmi	Korean	1996	Bacillus subtilis duplex viable granules	*Enterococcus Faecium* *&* *Bacillus subtilis*
Life-Space	Australia	2012	Probiotics Powder for Infant	*Lactobacillus fermentum*
BioGaia	Sweden	1990	Protectis	*Lactobacillus reuteri*
Swisse	Australia	1969	DAILY DIGESTIVE PROBIOTIC	*Lactobacillus acidophilus*
GNC	USA	1935	Probiotic complex	*Lactobacillus acidophilus*
Hyperbiotics	USA	2006	Hyperbiotics PRO-15	*Lactobacillus reuteri* *&* *Lactobacillus paracasei*
CHR. Hansen	Denmark	1874	Puractive	*Lactobacillus rhamnosus* GR-1

The manufacturers in this table are ranked in no particular order.

## 3 Causes of hypertension

### 3.1 Age

The incidence of hypertension was highest in individuals aged over 40 and increased with age (Wang et al., 2020). According to an analysis of Statistics Canada statistics, the incidence of high blood pressure is positively correlated with increasing age (DeGuire et al., 2019). A national survey on hypertension in France in 2015 found that hypertension was prevalent among individuals aged 18 to 74, with the incidence in men being slightly higher than that in women (Vallée et al., 2020). For middle-aged hypertensive patients aged 30 to 50 years, the manifestation was increased diastolic blood pressure, along with an increase in systolic blood pressure. Simple diastolic hypertension was more common in middle-aged men. Weight gain was associated with increased peripheral vascular resistance and normal cardiac output. For elderly hypertension patients aged over 65 years, simple systolic hypertension was the most common type (Briasoulis et al., 2014). Epidemiology has demonstrated that systolic blood pressure increases with age, while diastolic blood pressure gradually decreases after 55 years of age (Jennings et al., 2019).

### 3.2 Eating habit

People who consume excessive amounts of salt have been found to have an increased incidence of hypertension (He et al., 2020). High-sodium diets have been linked to changes in various proteins involved in calcium stabilization and heart muscle contraction, and excessive sodium intake has been associated with hypertension, stroke, and various cardiovascular diseases. Notably, the salt-sensitive population demonstrates an unusually sensitive renal response to salt intake. This is due to the abnormal overreaction of the sympathetic nervous system, coupled with weakened inhibition of the renin-angiotensin axis, which results in the blocking of nitric oxide (NO) synthesis by endothelial cells, leading to increased vascular resistance and hypertension in the salt-sensitive population. Poor dietary habits are a major cause of hypertension. A study on five dietary patterns for middle-aged and elderly people in the rural areas of Beijing, Northern China, revealed that an irrational dietary pattern characterized by high consumption of refined wheat, meat, poultry and alcoholic beverages was positively correlated with blood pressure (Li et al., 2023).

### 3.3 Obesity

**Figure 3:**
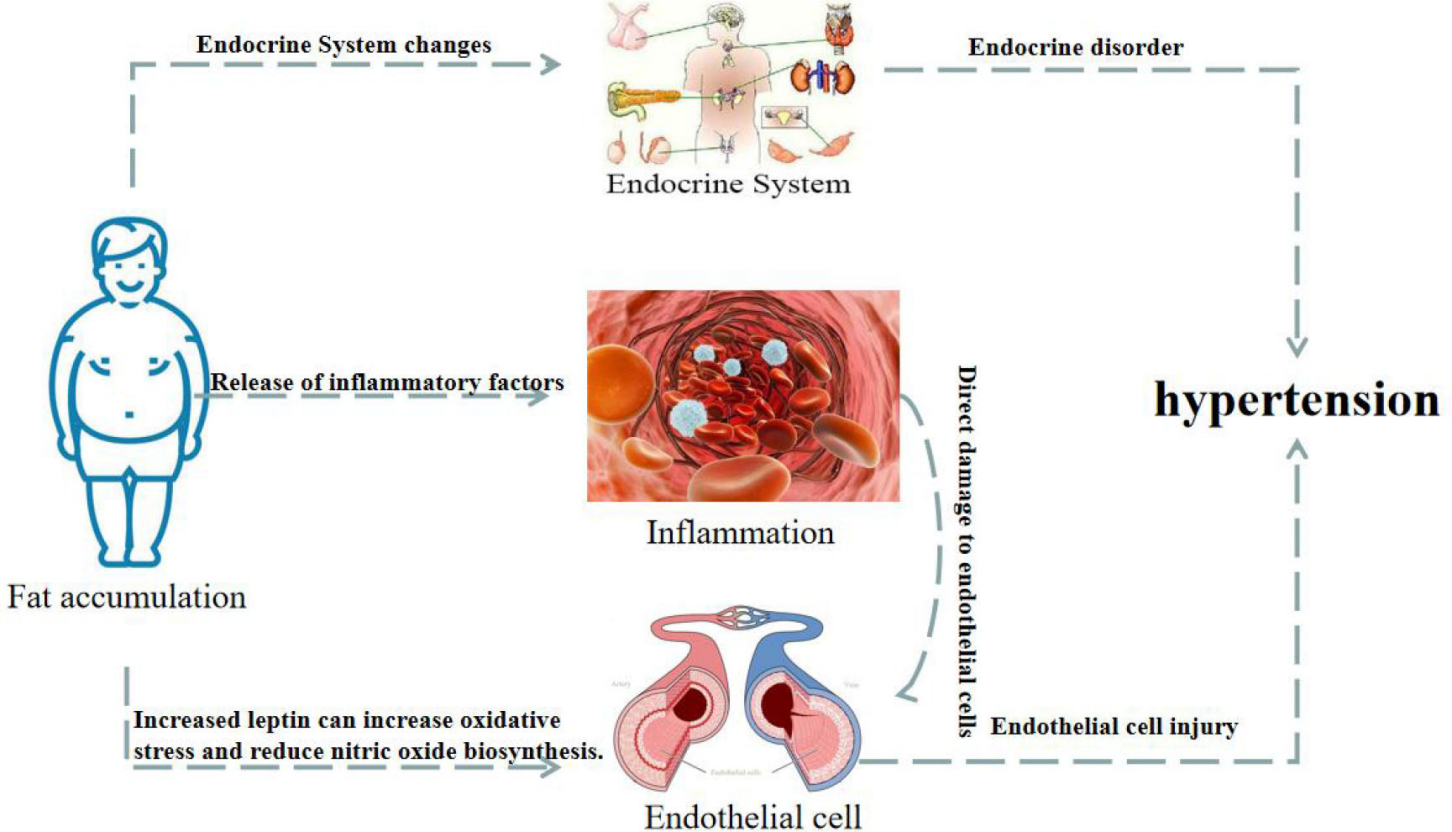
Changes of endocrine, vascular inflammation and endothelial cells in obese patients lead to hypertension.

Obesity is associated with an increased incidence of hypertension. As shown in **Figure 3**, this can be attributed to several changes in endocrine, inflammatory, and endothelial cell levels (Seravalle and Grassi, 2017; Xiao and Harrison, 2020; Cober et al., 2022; Fernandes-Rosa et al., 2023). Adolescent primary hypertension is largely driven by the increase in visceral fat deposition, which triggers the typical biological maturation and metabolic abnormal syndrome, and ultimately leads to the release of adrenaline (Manosroi and Williams, 2019). The mechanisms underlying obesity-related hypertension include the release of leptin, adipokines, and inflammatory factors, which promote vascular inflammation, damage endothelial function, and contribute to the proliferation, migration and foam cell formation of vascular smooth muscle cells (VSMC), platelet adhesion and aggregation, and atherosclerosis, resulting in elevated blood pressure (Supriya et al., 2018; Recinella et al., 2020).

Hyperleptinemia and hypoadiponectinemia are common among obese hypertensive patients (Perrotta et al., 2019; Gruber et al., 2021). Elevated leptin levels have been found to increase oxidative stress, reduce NO biosynthesis, and promote sodium reabsorption, resulting in increased arterial blood pressure (Bełtowski, 2006). On the other hand, adiponectin has been shown to have a protective effect on the cardiovascular system, increasing insulin sensitivity and inhibiting inflammation (Cohen et al., 2022). The plasma adiponectin levels were also found to be negatively correlated with blood pressure. Furthermore, most of the body is covered with fat outside of the blood vessels. This adipose tissue can release lipid-derived vasodilator factors to act on the potassium channels of the vascular smooth muscle, causing vasodilation. However, this lipid-derived vasodilation response is impaired in hypertension. In one study, adipose tissue and VSMC were co-cultured, and the expression of angiotension II (Ang II) receptor and RhoA and Rho kinases were found to be increased in the VSMC. These signaling molecules are known to play an important role in vascular remodeling and blood pressure regulation (Savoia et al., 2005). Moreover, when the area of the visceral fat was further increased, various metabolic disorders and cardio-renal vascular target organ damage began to emerge successively.

### 3.4 Heredity

Hypertension is a complex polygenic disorder, with many genes influencing its development (Zhao et al., 2013). If both parents have high blood pressure, their children are at higher risk of developing hypertension (Weijmans et al., 2015). Hypertension may be inherited through two different mechanisms: gene dominant inheritance and polygene association inheritance (Stoll et al., 2018; Teixeira et al., 2018). Advances in molecular biology have offered further insight into the pathogenesis of hypertension in these conditions, elucidating how several risk genes and environmental factors interact with each other and ultimately lead to hypertension (Kawarazaki and Fujita, 2021). In recent years, genome-wide association studies have identified more than 100 single nucleotide polymorphisms associated with blood pressure phenotypes and mutations in multiple single genes that lead to diseases with elevated blood pressure, such as glucocorticoid-inhibiting aldosteronism and Gordon syndrome (Padmanabhan et al., 2017; Mabillard and Sayer, 2019; Zennaro et al., 2020). Mutations in some genes are also associated with increased blood pressure (Matsuo et al., 2004; Dungan et al., 2009; Zhang et al., 2015; Fan et al., 2017). Further research into the genetic and DNA expression of hypertension may provide new pathways for blood pressure regulation and new ideas for hypothesized drug targets for hypertension.

### 3.5 External environment

Studies have found that people who live in noisy train stations or downtown areas have a higher risk of hypertension compared to those who reside in quiet, rural areas (Lee et al., 2019). This is due to the increase in norepinephrine secretion and increased heart rate caused by noise, as well as the reduction in blood magnesium, a protective agent for heart muscle, leading to a higher incidence of coronary heart disease (Münzel et al., 2017). Experimental evidence has demonstrated that in exposed noise environments, the walls of capillaries are more prone to deformation, which slows down blood flow and can damage both the myocardial and vascular systems. Hence, noise and other living environment factors can also significantly increase the risk of developing hypertension (Münzel et al., 2014; Hahad et al., 2019). High altitude and cold living environments are additional risk factors for hypertension (Shen et al., 2017; Chen and Sun, 2017).

## 4 Probiotic adjuvant therapy for hypertension

### 4.1 Routine clinical treatment

Numerous clinical trial data currently support the use of medication or low doses of clothiazone, angiotensin receptor blockers, and patient-specific therapy to reduce blood pressure. However, the efficacy of these new treatment strategies for patients with diabetes, chronic kidney disease, pregnancy, and other conditions remains to be further discussed. For example, in hypertensive women, ACE inhibitors and ARBs may need to be discontinued during pregnancy, and other options such as methyldopa and labetalol may be worth considering (Lu et al., 2018). In addition, many kinds of antihypertensive drugs have been found to have many side effects. The side effects of some common antihypertensive drugs are summarized in **Table 2** (Laurent, 2017).

**Table 2:** Several conventional hypertension drugs and their associated side effects.

Hypertension drugs	Side effects
diuretic	Electrolyte disorder, induce diabetes, increase cholesterol, glyceride and blood uric acid
β- receptor blocker	Induce bronchial asthma, abnormal lipid metabolism, aggravation of heart failure, mask hypoglycemia symptoms, and induce depression
ACE inhibitors	Cause cough, gastrointestinal dysfunction, liver dysfunction, angioneurotic edema
Calcium ion blocker	Cause headache, rapid heartbeat, edema, rash and allergic reaction
Ang II receptor inhibitor	Dizziness, hypotension, hyperkalemia, renal dysfunction, gastrointestinal dysfunction

### 4.2 Probiotics adjuvant therapy and the underlying mechanisms

**Figure 4:**
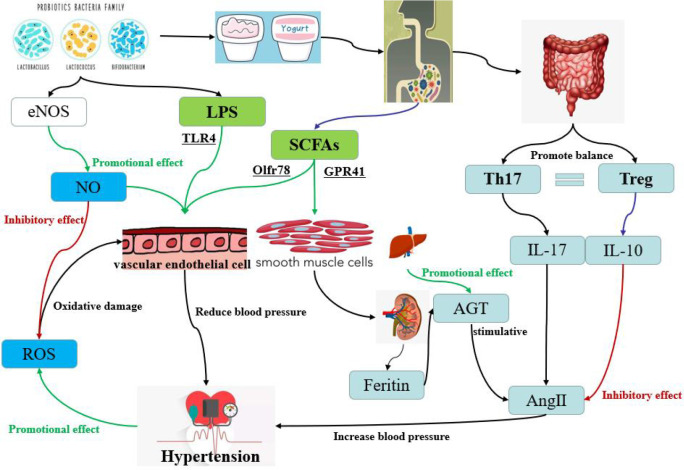
The different pathways of hypertension regulation by probiotics in yogurt, one of the common functional foods. The red pathway is inhibitory and the green pathway is accelerative. The pathways contain oxidative stress pathway, short-chain fatty acid pathway, dndothelial dysfunction pathway and bacterial LPS pathway.

Gut microbial richness and diversity have been linked to hypertension in both animals and humans, and dysregulation of the microbiome increases the risk of developing the condition (Yang et al., 2015; Li et al., 2017b). Clinical studies suggest that the use of probiotics can lead to moderate to significant reductions in blood pressure (Yuan et al., 2023). For instance, consuming beverages containing *Lactobacillus plantarum* for a period between 1-2 months has been found to reduce vascular systolic blood pressure, fibrinogen, and interleukin levels in healthy participants. Furthermore, several clinical trials for hypertension have reported that the use of *Lactobacillus*-fermented milk significantly reduced blood pressure (Aoyagi et al., 2017). In recent years, the mechanism of probiotics in hypertension has been the subject of many studies, and the impact of probiotics on blood pressure levels has been increasingly recognized. The studies related to probiotics in the treatment of hypertension are listed in **Table 3**. It is clear from these studies that the treatment of hypertension relies not only on a single probiotic, but also on a combination application of probiotics. Moreover, the rich types of functional foods also provide a variety of carriers for the probiotics. As shown in **Figure 4**, four probiotics are used as adjunctive therapy for hypertension and their mechanisms of action.

**Table 3:** Recent studies on probiotics in the treatment of hypertension.

Probiotics	Functional carrier	Experimental model	Principle of action	Treatment time	Reference
*Lactobacillus delbrueckii* Lb100 *&* *Lactococcus lactis* AM1	Yogurt	SHRs	ACE-Inhibitory	4 weeks	(Glazunova et al., 2022)
*Lactobacillus helveticus* H9	Fermented milk	SHRs	ACE-Inhibitory	6-12h	(Chen et al., 2014)
*Bifidobacterium bifidum* MF20/5	Fermented milk	\	ACE-Inhibitory	\	(Gonzalez-Gonzalez et al., 2013)
*Lactiplantibacillus plantarum* NK181 *& Lactobacillus delbrueckii* KU200171	Yogurt	\	Antioxidant and ACE-Inhibitory	\	(Kim et al., 2021)
*Lactiplantibacillus plantarum* SR37-3 & *Lactiplantibacillus plantarum* SR61-2	Fermented milk	SHRs	L-NAME inhibiting	4 weeks	(Yuan et al., 2022)
*Lactobacillus acidophilus* BCRC14065 *&* *Lactobacillus delbrueckii* subsp. *lactis* BCRC12256 *&* *Lactobacillus gasseri* BCRC14619	Yogurt	SHRs	Activating compensatory IGF-IR/PI3K/Akt survival pathways	8 weeks	(Lin et al., 2013a)
*Lactobacillus casei* Shirota	Fermented milk	Clinical Experiment	\	3 times a week	(Aoyagi et al., 2017).
*Lactobacillus* *helveticus* CM4	The tablets containing powdered fermented milk	Clinical Experiment	ACE-Inhibitory	4 weeks	(Aihara et al., 2005)
*Lactobacillus acidophilus* NCDC-15	Fermented camel milk	\	ACE-Inhibitory	\	(Solanki et al., 2022)
*Lactobacillus casei* ATCC7469	Fermented milk	\	ACE-Inhibitory	\	(Elkhtab et al., 2017)
*Lactobacillus plantarum* C4	Fermented milk	\	ACE-Inhibitory	\	(Moreno-Montoro et al., 2017)
*Lactobacillus bulgaricus* LB6	Fermented milk	\	ACE-Inhibitory	\	(Shu et al., 2019)
*Lactiplantibacillus plantarum* Y7	Fermented pickle	\	\	\	(Kim et al., 2022)
Kefir	Fermentation of milk by kefir grains	SHRs	Improving the structural and functional integrity of the intestinal wall and preventing neuroinflammation within the heart’s regulatory nucleus	9 weeks	(De Almeida Silva et al., 2020)
Kefir	Fermented milk	Male Wistar rats	ACE-Inhibitory	7 weeks	(Amorim et al., 2019)
*Lactobacillus helveticus* R0389 *&* *Lactocaseibacillus rhamnosus* R0011	Fermented milk	Cell model	ACE-Inhibitory& Inducing the production of NO	\	(Adams et al., 2020)
*Lactobacillus bulgaricus* *&* *Lactobacillus helveticus* MB2-1 *&* *Lactobacillus plantarum* B1-6 *&* *Lactobacillus plantarum* 70810	Navy bean milk	\	ACE-Inhibitory	\	(Rui et al., 2015)
*Lactobacillus acidophilus* BCRC14065 *&* *Lactobacillus delbrueckii* subsp. *lactis* BCRC12256 *&* *Lactobacillus gasseri* BCRC14619	Yogurt	SHRs	Inhibiting inflammatory pathways	\	(Lin et al., 2013b)
*Lactobacillus casei* SY13	Yogurt	\	ACE-Inhibitory	\	(Obaroakpo et al., 2019)
*Lactobacillus casei* BGP93 *&* *Streptococcus thermophilus* TA-40	Dairy beverages	\	ACE-Inhibitory	\	(Pereira et al., 2019)
*Lactobacillus* sp. FTDC2113 *&* *Lactobacillus acidophilus* FTDC8033 *&* *Lactobacillus acidophilus* ATCC*4356* *&* *Lactobacillus casei* ATCC*393* *&* *Bifidobacterium* FTDC8943 *&* *Bifidobacterium longum* FTDC8643	Soymilk	\	ACE-Inhibitory	\	(Yeo and Liong, 2010)
*Lactobacillus plantarum* DSM15313	Blueberries fermented	SHRs	\	4 weeks	(Ahrén et al., 2015)
*Clostridium butyricum pMTL007-GLP-1*	Engineered probiotics	SHRs	\	\	(Wang et al., 2023)

The table summarizes the probiotic strains, functional carriers of probiotics, experimental models, mechanisms of action, and duration of treatment. The “/” indicates that the relevant content is not mentioned in the reference.

#### 4.2.1 Improvement of vascular oxidative stress

Oxidative stress is a pathological process in which the production of reactive oxygen species (ROS) is excessive or the antioxidant capacity of body is weakened, resulting in the accumulation of ROS and the oxidative damage of cells (Senoner and Dichtl, 2019). Hypertension has been closely linked to oxidative stress (Guzik and Touyz, 2017). When blood vessels are damaged, their systolic and diastolic dysfunction is a direct cause of abnormal blood pressure. Endothelial NO synthase (eNOS) is a key enzyme in the synthesis of NO by the vascular endothelium. The NO produced by eNOS can inhibit the production of ROS and protect the vascular endothelium (Kim et al., 2020). However, eNOS receptors are regulated by various proteins such as fovetin, calmodulin, heat shock protein and Ang II (Rochette et al., 2013). Friques et al. (2015) found that probiotic kefir can maintain blood pressure stability by restoring the balance between ROS and NO, but the treatment time for kefir must last for about 60 days. At the same time, Ang II can also activate the production of ROS (Montezano et al., 2014). Furthermore, in cases of hypertension, the further increase of ROS activity leads to endothelial dysfunction, smooth muscle cell proliferation, lipid oxidation, inflammation, and other occurrences, all of which contribute to vascular damage (Incalza et al., 2018). Wu et al. (2021) revealed for the first time the antioxidant and vascular remodeling-reducing effect of RND3 in hypertensive rats and its molecular mechanism. They found that RND3 regulated superoxide production, vascular smooth muscle cell migration and proliferation, and blood pressure vessel remodeling by inhibiting RICK1-NOX1/2 and mitochondrial superoxide signaling.

For the antihypertensive effect of the probiotic *Lactobacillus fermentum* CECT5716 (LC40) through the NO system, Robles-Vera et al. (2018) constructed a common model of hypertension (N^G^-nitro-L-arginine methyl ester added to drinking water). In this model, *Bifidobacterium* decreased significantly. After the addition of LC40 and N^G^-nitro-L-arginine methyl ester, *Bifidobacterium* did not decrease significantly, and *Propionibacterium* recovered to its normal level.The probiotic kefir mentioned earlier has a regulatory effect on the balance between NO and ROS, while Azizi et al. (2021) also provided a detailed summary of the therapeutic examples of this probiotic to date in his review, including kefir’s special functions of antioxidant and antihypertensive properties.

#### 4.2.2 Production of short chain fatty acids

SCFAs play an integral role in both health maintenance and disease development. SCFAs have been shown to protect the heart and blood vessels from vascular damage (Bartolomaeus et al., 2019). Composed of organic fatty acids with a carbon atomic number between 2 and 6, SCFAs are consisted primarily of acetic acid, propionic acid, butyric acid, isobutyric acid, isovaleric acid and valeric acid. SCFAs not only provide energy as a nutrient, but also have important physiological regulatory roles, such as controlling cell proliferation and differentiation, apoptosis, immune response, nutrient absorption and lipid metabolism. Robles-Vera et al. (2020b) fed LC40 and *Bifidobacterium brevis* CECT7263 (BMF) directly to mice. Chronic treatment with LC40 or BFM increases the bacteria that produce butyrate and prevents an increase in blood pressure in SHRs. Oral therapy with either butyrate or acetate also prevents an increase in blood pressure and F/B ratio, while restoring the balance of type 17 helper T cells (Th17) and regulatory T cells (Tregs) in the MLN.

The prevalence of hypertension is largely linked to the intake of foods with different fiber content. What we eat every day has a great impact on the structure and metabolism of gut microbes, such as metabolites such as TMAO and SCFAs, which are key factors in gastrointestinal diseases, cancers, and cardiovascular diseases, particularly hypertension (Verhaar et al., 2020). The SCFAs produced by microbial fermentation of dietary fiber and resistant starch in the cecum and colon, can reduce the risk of ecological disorders in patients with hereditary hypertension and prevent the development of hypertension (Robles-Vera et al., 2020a). SCFAs pass the epithelial mucosal barrier in the form of ionization through 1:1 exchange with bicarbonate, hydrogen-coupled monocarboxylate transporter 1 (MCT-1), MCT-2, MCT-4, and sodium-coupled monocarboxylate transporter 1 (SMCT-1). It performs its physiological role through two mechanisms: the first is to acetylate lysine residues by inhibiting histone deacetylases (HDACs) and promote the binding of transcription factors to promoter regions to regulate gene expression (Luu et al., 2020); the second is to perform its biological function by binding to G protein coupled receptors (GPCRs) (Parada Venegas et al., 2019).

Four types of GPCRs have been identified that are activated by SCFAs, including GPR41/FFAR3, GPR43/FFAR2, GPR109A, and Olfr78 (Pluznick et al., 2013; Kimura et al., 2020; Li et al., 2020). These receptors are widely expressed in intestinal epithelial cells, adipocytes, immune system, vascular smooth muscle cells, and vascular endothelial cells. It has been found that SCFAs are mainly involved in the regulation of blood pressure through Olfr78 and GPR41 (Pluznick et al., 2013; Pluznick, 2017). Olfr78 is mainly distributed in the smooth muscle cells of renal entry arterioles and peripheral resistance vessels, and GPR41 is distributed in the endothelial cells of blood vessels. Pluznick et al. (2013) have discovered that Olfr78 is expressed in the kidneys and mediates renin secretion in response to SCFAs, specifically in response to propionate. Specifically, renin can play a promoting role in the generation of Ang II in plasma (Patel et al., 2017). Mice deprived of Olfr78 were particularly sensitive to a large and rapid drop in blood pressure when given propionate, suggesting that the normal function of Olfr78 is to raise blood pressure and counter the hypotensive effects of SCFAs. In contrast, mice lacking the GPR41 coding gene did not respond to propionate hypotension, and this dose resulted in a strong hypotensive response in wild-type mice, suggesting that GPR41 was involved in lowering blood pressure in response to propionate. Natarajan et al. (2016) confirmed that GPR41 can reduce blood pressure by affecting vascular tension. Reducing the gut microbiome of Olfr78 knockout mice by giving them antibiotics led to an increase in their blood pressure, suggesting that propionate produced by the gut microbiome regulates blood pressure through the Olfr78 receptor (Pluznick, 2014). Bartolomaeus et al. (2019) also found that long-term administration of propionate could alleviate endothelial dysfunction and thus inhibit blood pressure fluctuation.

#### 4.2.3 Repair of endothelial cell dysfunction

Vascular endothelial cell (VEC) dysfunction is closely related to hypertension, as it plays an important role in the occurrence and progression of hypertension. Furthermore, hypertension itself can exacerbate VEC dysfunction, resulting in a vicious cycle. VECs are a continuous layer of flat cells that line the interior and exterior walls of the blood vessels, providing a smooth surface for blood flow and maintaining normal blood flow. Recent studies have revealed that VECs also have both endocrine and paracrine functions, secreting various vasoactive substances, such as vasodilator factors and contractile factors, that help maintain vascular wall tension (Oruc et al., 2017).

VECs are metabolically active cells, and hypertension stimulates their secretion of various physiological regulatory factors, the most important of which are the regulation of shear stress and pulsatile blood flow. Systolic blood pressure reflects the shear stress of blood flow on the vascular wall, while pulse pressure reflects both shear stress and the pulsatility of blood flow, both of which contribute to regulating VEC secretion and leading to endothelial dysfunction (Tesauro et al., 2017). Additionally, the ischemia and hypoxia caused by vasospasm and contraction, which are common in hypertension, further damage endothelial function and lead to changes that are characterized by endothelial dependence and weakened diastolic response (Lüscher et al., 1993). Hypertension stimulates VECs to produce and release a series of endothelial factors, causing smooth muscle cell (SMC) proliferation and hypertrophy, intima collagen deposition, and thickening of the vessel walls, thus increasing peripheral vascular resistance and further aggravating hypertension (Schober and Zernecke, 2007).

Rashid et al. (2014) have evaluated the potential of probiotics to resist hypertension in rats. This study ultimately showed that the consumption of probiotic VSL3 solution can prevent the endothelial dysfunction of Mesentery artery in rats, presumably by reducing bacterial migration and local angiotensin system activity, thereby improving oxidative stress and preventing hypertension. Malik et al. (2018)conducted a six-week clinical study using probiotic *Lactobacillus plantarum* 299v as a supplement and found that this probiotic can improve endothelial dysfunction and also reduce systemic inflammation. Toral et al. (2014) tested the probiotic *Lactobacillus coryniformis* CECT5711 to reduce inflammation in mice, and found that long-term feeding of probiotics can also improve endothelial dysfunction and vascular oxidative stress.

#### 4.2.4 Reduction of vascular inflammation

Chronic inflammatory response is closely related to the occurrence and development of vascular diseases (Pugh and Dhaun, 2021). And a long-term low-activity micro-inflammatory state of the body is the most prominent sign of dysfunction of vascular endothelial cells (Engin, 2017). Current studies have shown that long-term hypertension in patients can lead to damage of vascular endothelial function, subsequently leading to more serious cardiovascular and cerebrovascular diseases (Sitia et al., 2010). Animal studies demonstrated elevated levels of inflammatory factors in the plasma and walls of blood vessels in hypertensive animals (Murray et al., 2021). Epidemiological and clinical trials have shown that plasma inflammatory factor levels in hypertensive patients are significantly higher than those in people with normal blood pressure (Yi et al., 2022). Atherosclerosis, a type of chronic inflammation of blood vessels, is linked to hypertension, with some studies suggesting that inflammation and hypertension can both influence each other. Inflammation can promote the onset of hypertension by altering the biological activity of NO and reducing endotheloid-dependent vasodilator factors, leading to increased expression of C-reactive protein, tumor necrosis factor, cell adhesion molecules, chemokines, growth factors, endothelin (ET), angiotensin, and other inflammatory factors (Soehnlein and Libby, 2021; Mahdavi-Roshan et al., 2022). Vascular endothelial cells are the direct and primary site of damage in vascular inflammation, and the occurrence of endothelial dysfunction will further lead to the worsening of vascular inflammation. In a review article, Grylls et al. (2021) introduced that the action mechanism of microbiome on host hypertension may be the effect of its component lipopolysaccharide (LPS). Vasodilation, vascular inflammation, and hypertension were improved after probiotics supplementation, with these changes being attributed to LPS activation of endothelial toll-like receptor 4 (TLR4) in probiotics (Lin et al., 2006; Suadoni, 2021). At the same time, a series of signaling pathways associated with endothelial cells, including oxidase- ROS, eNOS-NO, and NADPH oxidase pathways, as well as vascular inflammatory signaling pathways involving mitogen activated protein kinase (MAPK) and nuclear factor kappaB (NF-kB), were also activated simultaneously.

Long-term use of LC40 could treat and prevent intestinal flora disorders, and restore the balance of altered helper Th17 and Tregs in mesenteric lymph nodes (MLN) (Toral et al., 2018). Th17 cells produce interleukin-17 (IL-17), which has powerful pro-inflammatory effects, and the IL-17 content in the plasma of hypertensive patients remains at a high level (Zhang et al., 2019). Moreover, IL-17 is associated with Ang II, which is an important mediator of Ang II-induced hypertension (Basile et al., 2021). In contrast to Th17, Tregs regulate hypertension (Barhoumi et al., 2011). The anti-inflammatory factor IL-10, produced by Tregs, can effectively improve hypertension. Kassan et al. (2011) provided evidence that Tregs played a protective role through IL-10, demonstrating its ability to slightly improve the endothelium-dependent vasodilator response to hypertension. Tinsley et al. (2010) found that daily intraperitoneal injection of recombinant IL-10 restored normal blood pressure in pregnant rats with pregnancy-induced hypertension. Notably, IL-10 is known to have a vascular protection function, which can limit the vascular injury caused by Ang II (Barhoumi et al., 2011).

## 5 Future outlook

Increasing evidence suggests that probiotics play a critical role in regulating blood pressure, and may become a major force in the prevention and treatment of hypertension. This is because probiotics possess the function which is lack in clinical drugs for hypertension. A growing of researches suggest that regular consumption of probiotics, contained in yogurt, fermented milk and cheese, and in probiotic supplements, can help reduce hypertension and maintain healthy blood pressure levels as part of a healthy lifestyle. The treatment of probiotics is based on the principle of coordination and has certain specificity. Although it does not have the immediate effect of common hypertension drugs, probiotics can treat hypertension for a long time without side effects. Probiotics act beneficially by improving the microbial balance of the gut, promoting diversity, stability, and remodeling of gut microbiota, regulating the body’s inflammatory state, and enhancing the intestinal mucosal barrier function. However, there is no quick fix - research suggests that it takes a minimum of eight weeks of continuous probiotic consumption to reduce blood pressure, indicating that hypertension can be managed through diet, but it requires a long-term commitment. Nevertheless, this does not mean that probiotics can not play a role in preventing hypertension. In conclusion, using probiotics in the long-term prevention and treatment of hypertension provides a new, feasible idea for reducing the incidence of hypertension. Additionally, paying attention to dietary habits and living a healthy lifestyle are also measures to prevent and treat hypertension. The occurrence of hypertension is multi-factorial and multi-angle, but probiotics can regulate various mechanisms of hypertension through the gut microbiota, providing a new measure for hypertension.

## Author contributions

ZC: writing—original draft preparation, writing—review and editing, validation. WL: methodology; software. JL: methodology; software. JD: methodology. FG: methodology; software. DZ: methodology. TW: Conceptualization, validation, writing—review and editing, supervision, funding acquisition. ZX: Conceptualization, validation, writing—review and editing, supervision, funding acquisition. All authors contributed to the article and approved the submitted version.
